# The Complete Chloroplast Genome Sequences of Eight *Fagopyrum* Species: Insights Into Genome Evolution and Phylogenetic Relationships

**DOI:** 10.3389/fpls.2021.799904

**Published:** 2021-12-15

**Authors:** Yu Fan, Ya’nan Jin, Mengqi Ding, Yu Tang, Jianping Cheng, Kaixuan Zhang, Meiliang Zhou

**Affiliations:** ^1^College of Agriculture, Guizhou University, Guiyang, China; ^2^Institute of Crop Sciences, Chinese Academy of Agricultural Sciences, Beijing, China; ^3^College of Life Sciences and Food Engineering, Inner Mongolia MINZU University, Tongliao, China; ^4^College of Food Science and Technology, Sichuan Tourism University, Chengdu, China

**Keywords:** *Fagopyrum*, Polygonaceae, chloroplast genome, comparative analysis, phylogenetic relationship

## Abstract

Buckwheat (*Fagopyrum* genus, Polygonaceae), is an annual or perennial, herbaceous or semi-shrub dicotyledonous plant. There are mainly three cultivated buckwheat species, common buckwheat (*Fagopyrum esculentum)* is widely cultivated in Asia, Europe, and America, while Tartary buckwheat (*F. tataricum*) and *F. cymosum* (also known as *F. dibotrys*) are mainly cultivated in China. The genus *Fagopyrum* is taxonomically confusing due to the complex phenotypes of different *Fagopyrum* species. In this study, the chloroplast (cp) genomes of three *Fagopyrum* species, *F. longistylum*, *F. leptopodum*, *F. urophyllum*, were sequenced, and five published cp genomes of *Fagopyrum* were retrieved for comparative analyses. We determined the sequence differentiation, repeated sequences of the cp genomes, and the phylogeny of *Fagopyrum* species. The eight cp genomes ranged, gene number, gene order, and GC content were presented. Most of variations of *Fagopyrum* species cp genomes existed in the LSC and SSC regions. Among eight *Fagopyrum* chloroplast genomes, six variable regions (*ndhF*-*rpl32*, *trnS-trnG*, *trnC*, *trnE-trnT*, *psbD*, and *trnV*) were detected as promising DNA barcodes. In addition, a total of 66 different SSR (simple sequence repeats) types were found in the eight *Fagopyrum* species, ranging from 8 to 16 bp. Interestingly, many SSRs showed significant differences especially in some photosystem genes, which provided valuable information for understanding the differences in light adaptation among different *Fagopyrum* species. Genus *Fagopyrum* has shown a typical branch that is distinguished from the *Rumex*, *Rheum*, and *Reynoutria*, which supports the unique taxonomic status in *Fagopyrum* among the Polygonaceae. In addition, phylogenetic analysis based on the cp genomes strongly supported the division of eight *Fagopyrum* species into two independent evolutionary directions, suggesting that the separation of cymosum group and urophyllum group may be earlier than the flower type differentiation in *Fagopyrum* plants. The results of the chloroplast-based phylogenetic tree were further supported by the *matK* and Internal Transcribed Spacer (ITS) sequences of 17 *Fagopyrum* species, which may help to further anchor the taxonomic status of other members in the urophyllum group in *Fagopyrum*. This study provides valuable information and high-quality cp genomes for identifying species and evolutionary analysis for future *Fagopyrum* research.

## Introduction

As the organelle specialized for carrying out photosynthesis in plants, the chloroplast is descended from cyanobacteria, and occurs in eukaryotic autotrophs such as land plants and algae (Jin and Daniell, 2015; Gao et al., 2019). Chloroplasts are involved in photosynthesis and important biochemical processes including storage of starch, and the biosynthesis of sugars, several amino acids, lipids, vitamins, and pigments within plant cells, as well as sulfate reduction and nitrogen cycle supplying for the driving force of plants growth and development (Neuhaus and Emes, 2000; Jarvis and Soll, 2001; Leister, 2003; Bausher et al., 2006). As the center of photosynthesis, chloroplast has a complete genetic system, in which the genetic material is the cp genome (Zhao et al., 2019). Like nuclear DNA, chloroplasts have the same functions of replication, transcription, and inheritance, and cp genomes in plants are generally 10–20% of total genomes with an average length of about 120–170 kb (kilo-base pair) in tetrad ring structure (Shinozaki et al., 1986; Ruhlman and Jansen, 2014). The average cp genome size of land plants is 151 kb, with most species ranging from 130–170 kb in length, as well as the average GC content is 36.3%. The circle cp genome was separated by two inverted repeats (IRs, 20–28 kb) generating the large single copy (LSC, 16–27 kb) and the small single copy (SSC) (Jansen et al., 2007), which can provide abundant information for solving plant phylogenetic relationships and trends. Gene contents and sequences of cp genomes of angiosperm are generally conserved including 4 rRNAs, 30 tRNAs, and 80 unique proteins (Chumley et al., 2006). With the characteristics of parthenogenetic inheritance (maternal inheritance), relatively small genome and slow genome mutation rate (Palmer et al., 1988), analysis of the phylogenetic relationships of multiple chloroplast DNA can help to understand plant phylogeny, population genetic analysis, and taxonomic status at the molecular level (Alwadani et al., 2019). Although cp genomes of angiosperms are generally conserved in gene numbers and sequences (Jansen and Ruhlman, 2012), levels of structural variation in the genome different from various families and genera existed, such as gene duplication and large-scale rearrangement of genes, introns, and IR domains (Cosner et al., 2004; Lee et al., 2007; Cai et al., 2008; Guisinger et al., 2010; Martin et al., 2014).

The size of the cp genome was correlated with plant habits, environments, and other functional traits (Beaulieu et al., 2008; Li et al., 2018), making it a promising tool in studies of phylogeny, evolution, and population genetics of angiosperms (Tonti-Filippini et al., 2017). For example, the phylogenetic relationships among the main branches of flowering angiosperms were analyzed by using the coding genes from 64 cp genomes in *Amborella Baill* (Jansen et al., 2007); moreover, the relationship between genome evolution and phylogeny of *Zingiberaceae* was identified using the complete genome sequences of 14 chloroplasts of *Curcuma* Species (Liang et al., 2020).

*Fagopyrum* genus belongs to the Polygonaceae family, which are annual or perennial herb or semi-shrub plants (Zhang et al., 2021a). Wild buckwheats are mainly distributed in the regions of southwest China, which was recognized as the center of buckwheat origin and diversity (Ohnishi, 1995, 1998; Ohsako et al., 2002; Saski et al., 2005; Tang et al., 2010; Shao et al., 2011; Zhou et al., 2018). In 1742, *Fagopyrum* was established by Tourn, and named *Fagopyrum* Tourn ex Hall (Linnaeus, 1753). In 1992, the taxonomic status of buckwheat was confirmed, and the embryo position, morphology of cotyledon and perianth segments, characteristics of the pollen grain, and the basic number of chromosomes were taken as the basis for distinguishing *Fagopyrum* from *Polygonum* (Ye and Guo, 1992). With the continuous introduction of various buckwheat species, the classification based on morphological features gradually complicated, and plants from *Fagopyrum* were classified into 22–28 different species comprising two variants and two subspecies until 2021 (Zhang et al., 2021a). Due to the long-term change of buckwheat classification status, a consistent view of buckwheat was scarce, which limited the utilization of wild buckwheat varieties in plant improvement (Sharma and Jana, 2002; Neethirajan et al., 2011; Nagatomo et al., 2014). The controversies on buckwheat classification were including but were not limited to the following: (1) the genetic relationships among *F. tataricum*, *F. esculentum, F. esculentum* subsp. *ancestrale*, and *F. cymosum*. (2) The evolutionary paths between the cymosum group and urophyllum group are intersected or separated? (3) How to define the taxonomic status and phylogenetic relationship among *Fagopyrum* species in urophyllum group?

The rapid development of molecular biology and genomics provides favorable conditions for the study of cp genome of buckwheat plants, as well as the important genetic information for taxonomic status, phylogeny, and species identification. At present, five buckwheat cp genomes had been published, including *F. tataricum*, *F. esculentum*, *F. esculentum* subsp. *ancestrale*, *F. cymosum*, and *F. luojishanense* (Liu et al., 2008; Logacheva et al., 2008; Cho et al., 2015; Hou et al., 2015; Wang et al., 2017a; Zhang and Chen, 2018). However, the in-depth and conjoint study of *Fagopyrum* cp genome data sets was lacking, as well as the researches on buckwheat phylogeny and interspecific differences.

In this study, three cp genomes of *Fagopyrum* were sequenced, assembled, and annotated, then their cp genome data with five published ones were analyzed comprehensively, including characteristics of *Fagopyrum* cp genomes, codon usage, expansion of IR regions, SSRs analysis, and phylogenetic analysis of eight *Fagopyrum* species. Our objectives in this study were: (1) To present the complete sequence of cp genomes of three newly assembled buckwheat plants and to compare the global structure with five other previously published species (including one subspecies) within genus species comparisons; (2) SSR variations in the cp genome sequences of eight buckwheat plants were detected to develop a series of SSRs molecular markers that could be used to distinguish the relationship between different species; (3) The phylogenetic relationship and evolutionary path of buckwheat were reconstructed by combining genetic sequences based on eight cp genomes and six highly variable regions developed. (4) The taxonomic status of 17 buckwheat plants was discussed by using ITS and *matK* gene sequences.

## Materials and Methods

### Plant Material, Morphological Analysis, and DNA Extraction

In previous reports, we investigated in detail the survival status of *Fagopyrum* plants in southwest China (Cheng et al., 2020; Zhang et al., 2021a). The mature seeds of these plant materials are collected in the wild, then they are grown in the greenhouse of the institute of crop science, Chinese Academy of Agricultural Sciences (CAAS) in Beijing. The morphological details of eight *Fagopyrum* species were further observed. We mainly investigated the differences in plant type, leaf, inflorescence, seed and distribution (Cheng et al., 2020).

Further, the fresh leaves from three *Fagopyrum* species, *F. longistylum*, *F. leptopodum*, *F. urophyllum* were collected in Sichuan Province in 2020 (Supplementary Table 1). Voucher specimens of these samples were deposited in the Institute of Crop Sciences, Chinese Academy of Agricultural Sciences, Beijing, China. Total genomic DNA was isolated from 2 g of silica-dried leaf sample using the modified CTAB method (Doyle, 1987). In addition, we downloaded the available complete cp genomes of five other *Fagopyrum* species and three Polygonaceae species from GenBank [*F. tataricum*, MT712164.1; *F. cymosum* (*F. dibotrys*), KY275181.1; *F. esculentum*, MT364821.1; *F. esculentum* subsp. *ancestrale*, EU254477.1; *F. luojishanense*, KY275182.1; *Rumex hypogaeus*, MT017652.1; *Reynoutria japonica* (also known as *Polygonum cuspidatum*) MW411186.1; *Rheum officinale* MN564925.1] for phylogeny study.

### Genome Sequencing, Assembly, Annotation

The total DNA was disrupted by ultrasonic wave, and DNA libraries were read of 350 bp with purified DNA constructed by Library Prep Kit from NEBNext^®^. Total DNA was sequenced in Hiseq 4000 PE150. After filtering the low-quality data, raw sequencing data were checked and spliced using SPAdes 3.6.1 (Bankevich et al., 2012). Contigs were used to screen the cp genome by Blast Software, using published *F. esculentum* cp genome (MT364821) as the reference genome (Altschul et al., 1997). Selected contigs of the cp genome were assembled using Sequencher 4.10 Software (GeneCodes Corp., Ann Arbor, MI, United States), and all reads were mapped to validate cp genome using Geneious 8.1 Software (Kearse et al., 2012). Polymerase Chain Reaction (PCR) was done with specific primers of gaps, which were born after assembling genomes. The PCR products were sequenced by ABI 3730, and were involved in manually correcting annotations. The circular structure map was constructed by Organellar Genome DRAW^1^ (Lohse et al., 2013).

### Codon Usage Analysis

Codon Usage analysis was done by codonW 1.4.4 (Peden, 2000), and the values of relative synonymous codon usage (RSCU) were used to evaluate codon preference.

### Comparative Genomic Analysis

The divergence of 11 Polygonaceae genomes was counted by mVISTA in LAGAN mode (Frazer et al., 2004), and *Rumex hypogaeus* (MT017652), *Polygonum cuspidatum* (MW411186), and *Rheum officinale* (MN564925) were considered as the reference genomes. MAFFT was used to align all *Fagopyrum* species genome (Zhang et al., 2018), and the nucleotide diversity (Pi) of all complete cp genome was calculated using Launch DnaSP6 (Rozas et al., 2017), and the results were presented through a sliding window analysis with a window length of 600 bp and step size of 200 bp. Boundaries of inverted repeat (IR) regions, contraction, and expansion of eight cp genomes were determined using IRscope (Amiryousefi et al., 2018).

### Simple Sequence Repeats Analysis

To identify the microsatellites, the Perl script MISA70 and the SSRs parameter were used to analyze the SSRs detection based on the following conditions (Beier et al., 2017); thresholds were set as eight repeat units for mononucleotide SSRs, four repeat units for dinucleotide SSRs, four repeat units for trinucleotide SSRs, and three repeat units for tetranucleotide, pentanucleotide, and hexanucleotide SSRs.

### Phylogenetic Analysis

We used the 11 above-mentioned cp genomes to analyze the phylogenetic relationships among *Fagopyrum* species, including eight *Fagopyrum* species, and three Polygonaceae species (*Rumex hypogaeus*, *Rheum officinale*, and *Reynoutria japonica*) were used as outgroups. These cp sequences were aligned with the default parameters set using MAFFT program (Katoh and Standley, 2013) in GENEIOUS R8, and were manually adjusted in MEGA 6.0. The nucleotide sequence (*matK* and ITS) data were obtained from NCBI (Supplementary Table 9). The RAxML v7.2.8 program (Stamatakis, 2006) was used to perform the phylogenetic trees based on maximum likelihood analysis with 1000 bootstrap replicates. Bayesian inference was performed using the MrBayes v3.1.27 program (Ronquist and Huelsenbeck, 2003). Markov chain Monte Carlo simulations have two parallel runs with 2000,000 generations independently, and sampling trees every 100 generations. The initial 25% of trees were discarded as burn-in, and the remaining dates were used to construct a majority-rule consensus tree. Convergence diagnostics were monitored by examining the average standard deviation of split frequencies below 0.01.

## Results and Analysis

### Morphological Analysis in Eight *Fagopyrum* Species

**FIGURE 1:**
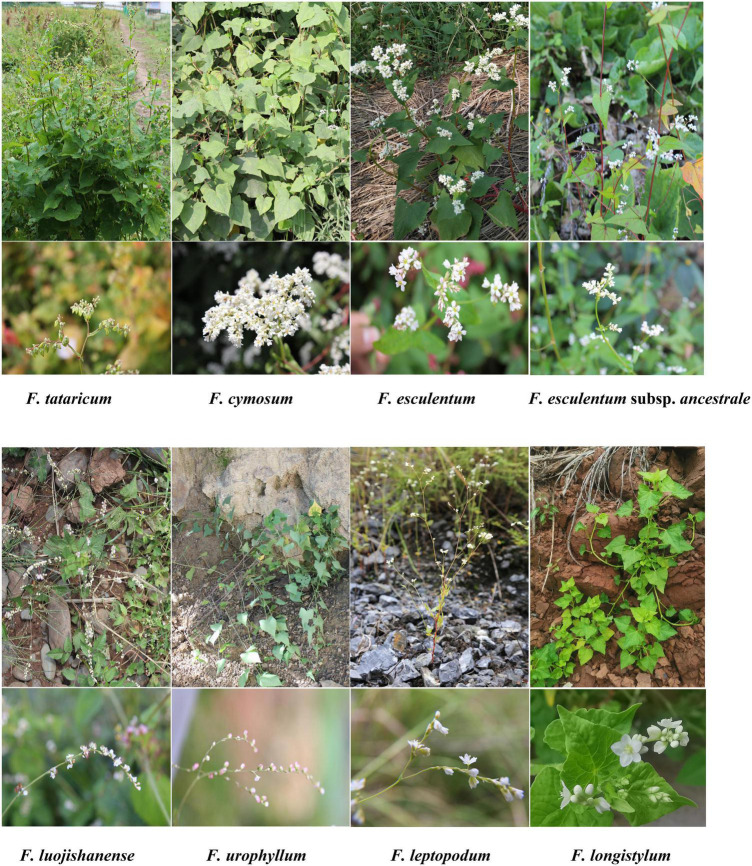
The morphological characters of plants and flowers of eight *Fagopyrum* species. **(A)** *F. tataricum*; **(B)** *F. cymosum*; **(C)** *F. esculentum*; **(D)** *F. esculentum* subsp. *ancestrale*; **(E)** *F. luojishanense*; **(F)** *F. urophyllum*; **(G)** *F. leptopodum*; **(H)** *F. longistylu*m.

The morphological characters of eight *Fagopyrum* species are further analyzed in this section. Buckwheat is a rare cereal crop that does not belong to Gramineae. *Fagopyrum* contains plants of both self-compatible (homostyly) and self-incompatible (heterostyly) species. Therefore, *Fagopyrum* species are good materials for studying the origin and spread of cultivated crops, as well as hot issues such as phylogenetic evolution of plants (Zhou et al., 2018). Morphological characteristics of eight typical different *Fagopyrum* species (including seven species and one subspecies) were systematically analyzed, and their differences were mainly concentrated in stems, leaves, flowers, and fruits (Figure 1 and Supplementary Table 1). In general, the morphology of *Fagopyrum* plants is relatively complex and their habits and features are various. In this study, three *Fagopyrum* species which cp genomes were not revealed were fully considered based on plant characteristics. *F. leptopodum*, which was commonly found in rocks and dry-hot valley areas, was considered to be a highly drought-resistant and barren resistant species. *F. longistylum*, a self-compatible but heteromorphic species, was a very rare phenomenon in plants. In addition, *F. urophyllum*, contained semi-woody branches and perennial rhizomes, which are considered as transitional species from herbaceous to woody plants (Ohnishi and Matsuoka, 1996; Zhang et al., 2021b).

### Characteristics of *Fagopyrum* Chloroplast Genomes

**FIGURE 2:**
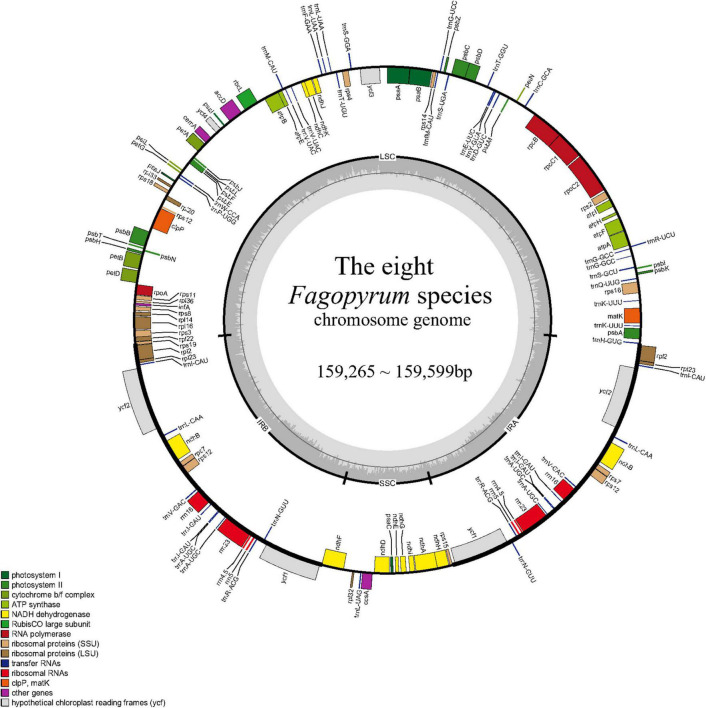
Gene map of the eight *Fagopyrum* species. The genes shown outside of the circle are transcribed clockwise, while those inside are transcribed counterclockwise. Genes belonging to different functional groups are color-coded. The dashed area in the inner circle indicates the GC content of the chloroplast genome.

The cp genomes of three wild *Fagopyrum* species were sequenced in this study, including two annual species (*F. longistylum* and *F. leptopodum*) and one perennial species (*F. urophyllum*). We obtained the complete cp genome sequences of 159,325 bp for *F. longistylum*, 159,350 bp for *F. urophyllum*, and 159,376 bp for *F. leptopodum*. Other published cp genomes of *Fagopyrum* were obtained from National Center for Biotechnology Information (NCBI), and all cp genomes ranged in size from 159,265 bp (*F. luojishanense*) to 159,599 bp (*F. esculentum* ssp. *ancestrale*) with 37.78–37.99% GC contents (Figure 2 and Table 1). Similar to other Polygonaceae, all cp genomes of cultivated and wild *Fagopyrum* species comprised a typical circular structure with four regions (Wu et al., 2020), and two inverted repeats (IR, IRa, and IRb) regions were separated by a LSC and a SSC (Figure 2). The LSC region in *Fagopyrum* accounted for 52.87–53.19% of the total cp genomes and ranged in size from 84,250 bp (*F. urophyllum*) to 84,885 bp (*F. esculentum* ssp. *ancestrale*); the SSC region in *Fagopyrum* accounted for 8.22–8.41% and ranged in size from 13,094 bp (*F. luojishanense*) to 13,406 bp (*F. urophyllum*); the *Fagopyrum* IR region accounted for 19.23–19.38% of the total size and ranged from 30,6845 bp (*F. esculentum* and *F. esculentum* ssp. *ancestrale*) to 30,870 bp (*F. luojishanense*). Moreover, the GC contents of all *Fagopyrum* cp genomes were similar, and the GC content of IR region was highest (41.26–41.48%), followed by the LSC region (36.01–36.32%) and the SSC region (31.97–32.99%).

**TABLE 1:** Comparison of the complete chloroplast genomes for eight *Fagopyrum* species.

		*F. tataricum*	*F. cymosum*	*F. esculentum*	*F. esculentum* ssp. *ancestrale*	*F. longistylum*	*F. leptopodum*	*F. urophyllum*	*F. luojishanense*
	Accession number	MT712164	KY275181	MT364821	EU254477	OK054489	OK054491	OK054490	KY275182
Total	Total length (bp)	159,272	159,320	159,576	159,599	159,325	159,376	159,350	159,265
	GC (%)	37.87	37.93	37.95	37.99	37.82	37.79	37.78	37.84
LSC	Length (bp)	84,397	84,422	84,875	84,885	84,417	84,282	84,250	84,431
	GC (%)	36.20	36.26	36.29	36.32	36.03	36.02	36.01	36.05
	Length (%)	52.99	52.99	53.19	53.19	52.98	52.88	52.87	53.01
SSC	Length (bp)	13,241	13,264	13,331	13,344	13,226	13,402	13,406	13,094
	GC (%)	32.78	32.90	32.99	32.96	32.17	32.10	31.97	32.36
	Length (%)	8.31	8.33	8.35	8.36	8.30	8.41	8.41	8.22
IRa/IRb	Length (bp)	30,817	30,817	30,685	30,685	30,841	30,846	30,847	30,870
	GC (%)	41.26	41.29	41.38	41.37	41.48	41.45	41.46	41.45
	Length (%)	19.35	19.34	19.23	19.23	19.36	19.35	19.36	19.38

There was little difference in coding regions in eight *Fagopyrum* species. Overall, they encode a total of 108–113 chloroplast genes, including 76–79 protein-coding genes, 28–30 tRNAs, and 4 rRNAs (Figure 2 and Table 2). All the above-mentioned genes were furtherly categorized as three parts, of which 47 genes belong to photosynthesis related genes (including rubisco, photosystem I, assembly/stability of photosystem I, photosystem II, ATP synthase, cytochrome b/f complex, cytochrome c synthesis, and NADPH dehydrogenase), 60 genes belong to transcription and translation related genes (including transcription, ribosomal proteins, and translation initiation factor, ribosomal RNA, and transfer RNA), and the remaining genes belong to biomacromolecule metabolism related genes or other unknown functions (Table 2). Moreover, among these various 113 genes, 15 genes contained one intron comprising 9 protein-coding genes (*atpF, petB, petD, ndhA, ndhB, rpoC1, rps12, rpl2*, and *rpl16*) and 6 tRNA genes (*trnA, trnG, trnI, trnK, trnL*, and *trnV*), while 2 genes (*ycf3*, *clpP*) contained two introns. In addition, *rps12* was identified as a noticeable trans-splicing gene of all *Fagopyrum* species, because the 5′ end of *rps12* exon was located in the LSC region but the other end of that was located in the IR domain.

**TABLE 2:** Genes contained in the chloroplast genome of eight *Fagopyrum* species.

Category for genes	Groups of genes	Name of genes
Photosynthesis related genes	Rubisco	*rbcL*
	Photosystem I	*psaA, psaB, psaC, psaI, psaJ*
	Assembly/stability of photosystem I	*ycf3*^a^*, ycf4*
	Photosystem II	*psbA, psbB, psbC, psbD, psbE, psbF, psbH, psbI, psbJ, psbK, psbL, psbM, psbN, psbT, psbZ*
	ATP synthase	*atpA, atpB, atpE, atpF*^a^*, atpH, atpI*
	Cytochrome b/f complex	*petA, petB*^a^*, petD*^a^*, petG, petL, petN*
	Cytochrome c synthesis	*ccsA*
	NADPH dehydrogenase	*ndhA*^a^*, ndhB*^a,b^*, ndhC, ndhD, ndhE, ndhF*^b^*, ndhK ndhG, ndhH, ndhI, ndhJ*
Transcription and translation related genes	Transcription	*rpoA, rpoB, rpoC1*^a^*, rpoC2*
	Ribosomal proteins	*rps2, rps3, rps4, rps7*^b^*, rps8, rps11, rps12*^a,b^*, rps14,rps15, rps16, rps18, rps19*^b^*, rpl2*^a,b^*, rpl14, rpl16*^a^*, rpl20, rpl22, rpl23, rpl32*^b^*, rpl33, rpl36*
	Translation initiation factor	*infA*
RNA genes	Ribosomal RNA	*rrn5*^b^*, rrn4.5*^b^*, rrn16*^b^*, rrn23*^b^**
	Transfer RNA	*trnA-UGC*^a,b^*, trnC-GCA, trnD-GUC, trnE-UUC, trnF-GAA, trnG-UCC, trnG-GCC*^a^*, trnH-GUG, trnI-CAU*^b^*, trnI-GAU*^a,b^*, trnK-UUU*^a^*, trnL-CAA*^b^*, trnL-UAA*^a^*, trnL-UAG, trnfM-CAUI, trnM-CAU, trnN-GUU*^b^*, trnP-UGG, trnQ-UUG, trnR-ACG*^b^*, trnR-UCU, trnS-GCU, trnS-GGA, trnS-UGA, trnT-GGU, trnT-UGU, trnV-GAC, trnV-UAC*^a,b^*, trnW-CCA, trnY-GUA*
Other genes	RNA processing	*matK*
	Carbon metabolism	*cemA*
	Fatty acid synthesis	*accD*
	Proteolysis	*clpP* *^a^*
Genes of unknown function	Conserved reading frames	*ycf1*^b^*, ycf2*^b^**
Pseudogenes		*ycf15*

*^a^Intron-containing genes.*

*^b^Genes located in the IR regions.*

### Codon Usage

Codon is the connection between the nucleic acids and proteins, and codon usage reflects the preference for selective use of codons encoding specific amino acids with genetic information (Wanga et al., 2021). The codon usage frequency of 79 protein-coding genes for 8 *Fagopyrum* species were calculated, and 64 codons were involved in encoding proteins containing three termination codons, such as UAA, UAG, and UGA (Table 3). The relative synonymous codon usage (RSCU) analysis showed that 30 codons of 8 *Fagopyrum* species were > 1, and the UUA encoding leucine had the highest RSCU with 1.85–1.87 in 8 *Fagopyrum* species. While the lowest RSCU was 0.33–0.36 with the CGC encoding arginine.

**TABLE 3:** Codon content of amino acids and stop codon of eight *Fagopyrum* species.

Amino acid	Codon	RSCUa																	
		*F. tataricum*	*F. cymosum*	*F. esculentum*	F. *esculentum* ssp. *ancestrale*	*F. longistylum*	*F. leptopodum*	*F. urophyllum*	*F. luojishanense*			*F. tataricum*	*F. cymosum*	*F. esculentum*	*F. esculentum* ssp. *ancestrale*	*F. longistylum*	*F. leptopodum*	*F. urophyllum*	*F. luojishanense*
Ala	GCA	1.12	1.13	1.14	1.11	1.16	1.16	1.16	1.15	Ile	AUA	0.97	0.97	0.97	0.96	0.97	0.97	0.98	0.98
	GCC	0.76	0.76	0.77	0.78	0.71	0.7	0.71	0.71		AUC	0.57	0.57	0.56	0.56	0.55	0.55	0.55	0.55
	GCG	0.48	0.48	0.47	0.47	0.49	0.5	0.49	0.49		AUU	1.46	1.46	1.47	1.48	1.48	1.48	1.47	1.48
	GCU	1.64	1.64	1.63	1.64	1.64	1.64	1.64	1.64	Lys	AAA	1.5	1.5	1.49	1.49	1.5	1.5	1.5	1.51
Arg	AGA	1.67	1.67	1.65	1.66	1.71	1.7	1.7	1.71		AAG	0.5	0.5	0.51	0.51	0.5	0.5	0.5	0.49
	AGG	0.75	0.75	0.77	0.76	0.73	0.74	0.73	0.73	Met	AUG	1	1	1	1	1	1	1	1
	CGA	1.45	1.45	1.48	1.48	1.46	1.47	1.45	1.45	Phe	UUC	0.65	0.66	0.67	0.67	0.66	0.65	0.65	0.66
	CGC	0.36	0.36	0.36	0.36	0.35	0.33	0.35	0.34		UUU	1.35	1.34	1.33	1.33	1.34	1.35	1.35	1.34
	CGG	0.45	0.44	0.43	0.45	0.43	0.44	0.45	0.43	Pro	CCA	1.08	1.08	1.06	1.08	1.08	1.09	1.08	1.08
	CGU	1.32	1.32	1.31	1.3	1.33	1.32	1.32	1.33		CCC	0.74	0.74	0.76	0.78	0.77	0.77	0.77	0.77
Asn	AAC	0.47	0.47	0.48	0.48	0.47	0.48	0.48	0.47		CCG	0.61	0.61	0.62	0.6	0.63	0.62	0.61	0.63
	AAU	1.53	1.53	1.52	1.52	1.53	1.52	1.52	1.53		CCU	1.57	1.57	1.56	1.54	1.53	1.52	1.54	1.53
Asp	GAC	0.42	0.42	0.42	0.42	0.41	0.41	0.41	0.42	Ser	AGC	0.43	0.43	0.44	0.44	0.42	0.42	0.43	0.43
	GAU	1.58	1.58	1.58	1.58	1.59	1.59	1.59	1.58		AGU	1.14	1.14	1.15	1.15	1.14	1.15	1.14	1.13
Cys	UGC	0.58	0.58	0.58	0.57	0.6	0.61	0.6	0.61		UCA	1.2	1.19	1.22	1.21	1.19	1.18	1.19	1.19
	UGU	1.42	1.42	1.42	1.43	1.4	1.39	1.4	1.39		UCC	0.94	0.95	0.94	0.94	0.96	0.97	0.95	0.96
Gln	CAA	1.53	1.53	1.52	1.51	1.52	1.52	1.51	1.52		UCG	0.65	0.66	0.63	0.62	0.64	0.65	0.65	0.64
	CAG	0.47	0.47	0.48	0.49	0.48	0.48	0.49	0.48		UCU	1.63	1.63	1.61	1.63	1.65	1.63	1.64	1.64
Glu	GAA	1.47	1.47	1.46	1.47	1.47	1.47	1.48	1.48	Thr	ACA	1.21	1.21	1.22	1.22	1.21	1.22	1.22	1.21
	GAG	0.53	0.53	0.54	0.53	0.53	0.53	0.52	0.52		ACC	0.74	0.75	0.74	0.73	0.71	0.71	0.71	0.71
Gly	GGA	1.53	1.52	1.52	1.52	1.57	1.57	1.57	1.57		ACG	0.54	0.53	0.53	0.53	0.58	0.56	0.57	0.58
	GGC	0.5	0.5	0.51	0.51	0.52	0.52	0.52	0.52		ACU	1.52	1.51	1.51	1.52	1.5	1.51	1.5	1.5
	GGG	0.74	0.75	0.75	0.74	0.69	0.69	0.7	0.69	Trp	UGG	1	1	1	1	1	1	1	1
	GGU	1.23	1.23	1.22	1.23	1.22	1.22	1.22	1.22	Tyr	UAC	0.44	0.44	0.42	0.41	0.42	0.42	0.42	0.42
His	CAC	0.47	0.47	0.47	0.47	0.47	0.47	0.48	0.47		UAU	1.56	1.56	1.58	1.59	1.58	1.58	1.58	1.58
	CAU	1.53	1.53	1.53	1.53	1.53	1.53	1.52	1.53	Val	GUA	1.41	1.41	1.41	1.41	1.39	1.41	1.41	1.4
Leu	CUA	0.85	0.84	0.85	0.84	0.84	0.85	0.85	0.84		GUC	0.58	0.58	0.58	0.59	0.59	0.58	0.58	0.58
	CUC	0.41	0.41	0.41	0.41	0.41	0.41	0.41	0.42		GUG	0.54	0.54	0.54	0.54	0.54	0.53	0.53	0.54
	CUG	0.38	0.38	0.41	0.4	0.42	0.41	0.41	0.42		GUU	1.46	1.47	1.46	1.46	1.48	1.48	1.48	1.48
	CUU	1.27	1.27	1.25	1.25	1.27	1.27	1.28	1.27	Stop	UAA	1.68	1.68	1.74	1.74	1.63	1.68	1.63	1.63
	UUA	1.87	1.87	1.86	1.87	1.86	1.86	1.85	1.85		UAG	0.74	0.74	0.63	0.63	0.79	0.74	0.79	0.74
	UUG	1.22	1.23	1.22	1.22	1.2	1.2	1.2	1.19		UGA	0.58	0.58	0.63	0.63	0.58	0.58	0.58	0.63

### Comparative Genomic Analysis

**FIGURE 3:**
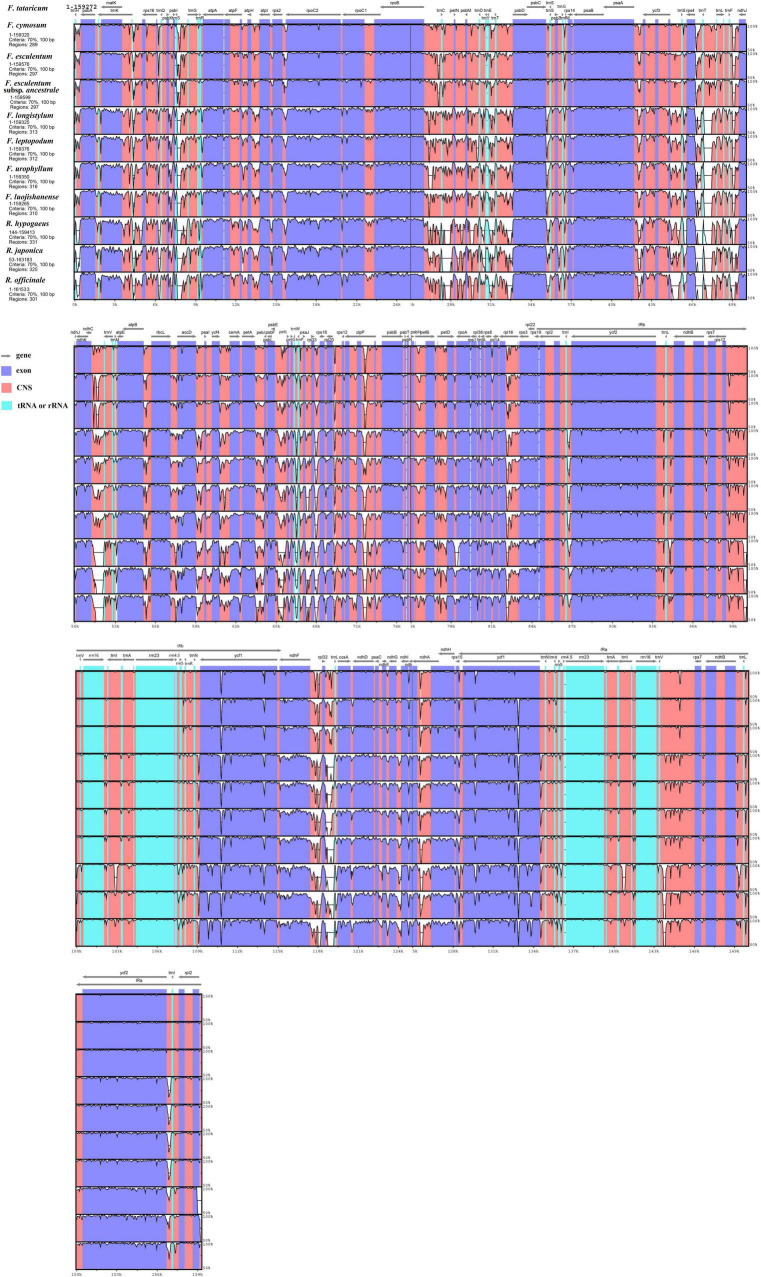
Sequence alignment of chloroplast genome among eight *Fagopyrum* species and three Polygonaceae species (*Rumex hypogaeus*, *Reynoutria japonica*, and *Rheum officinale*) with *F. tataricum* as a reference by using mVISTA. The Y-scale represents the percentage of identity ranging from 50 to 100%. Coding and non-coding regions are marked in purple and pink, respectively.

The genome of *F. tataricum* was served as the reference to conduct the mVISTA program for discovering *Fagopyrum* genome divergence, and three other genomes from Polygonaceae were regarded as the outgroups covering *Rumex hypogaeus*, *Polygonum cuspidatum*, and *Rheum officinale*. Results revealed that 11 cp genomes were relatively conserved (Figure 3). The three cultivated *Fagopyrum* species, four wild *Fagopyrum* species, and three outgroup members had higher similarity and low divergence, respectively. Furthermore, the divergence of LSC and SSC regions were higher than that of IR regions, and the non-coding regions exhibited greater variation than the coding regions.

**FIGURE 4:**
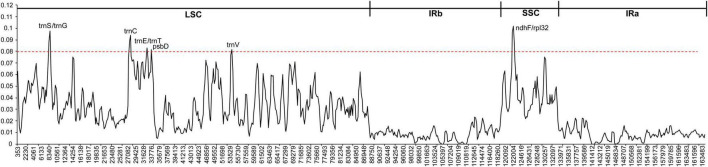
Comparison of nucleotide diversity (Pi) values among the eight *Fagopyrum* species. *X*-axis, position of the midpoint of each window; *Y*-axis, nucleotide diversity (Pi) of each window.

To further know the genetic diversity of various *Fagopyrum* species and exploit suitable polymorphic genes for identifying novel species, we calculate the nucleotide diversity (Pi) of eight *Fagopyrum* species. The Pi values were ranged from 0 to 0.10179 in the total cp genomes. The average Pi values of LSC and SSC regions were 0.0356 and 0.0445, respectively, but that of IR regions was 0.0084 (Supplementary Table 2). Most of the variations of *Fagopyrum* species cp genomes existed in the LSC and SSC regions. That is to say, two IR regions were more conserved than another two regions. A sliding window analysis showed that the Pi values of six regions were > 0.08, and these most divergent regions included *ndhF*-*rpl32*, *trnS*-*trnG*, *trnC*, *trnE-trnT*, *psbD*, and *trnV* (Figure 4 and Supplementary Table 2). Among them, three coding genes (*ndhF*, *rpl31*, and *psbD*) were highlighted, because coding genes were generally conserved. These polymorphic regions might be the critical loci for population genetic studies of *Fagopyrum* species.

### Contraction and Expansion of Inverted Repeats Regions Among Eight *Fagopyrum* Species

**FIGURE 5:**
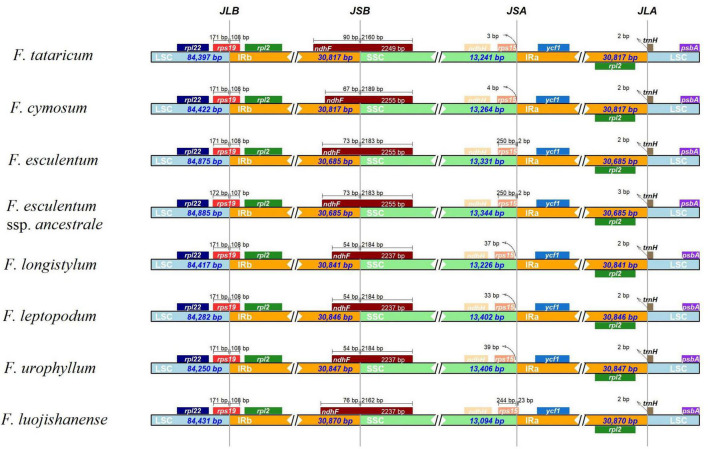
Comparison of the junctions between the LSC, SSC, and IR regions among eight *Fagopyrum* species chloroplast genomes. The figure is not scaled LSC, SSC, and IR.

As we all know, contraction and expansion of the IR regions are strongly linked to the length of cp genomes (Liang et al., 2020), therefore the IR boundaries were detected to explain the differences in *Fagopyrum* cp genome size. In general, IRs of wild *Fagopyrum* species (*F. longistylum*, *F. leptopodum*, *F. urophyllum*, and *F. luojishanense*) were longer than cultivated *Fagopyrum* species (*F. tataricum*, *F. cymosum*, and *F. esculentum*). Among them, the size of the IR regions of the two *F. esculentum* was the shortest (30,685 bp) and that of *F. luojishanense* was the longest (30,870 bp) (Figure 5).

Within the 8 *Fagopyrum* species, the *rps19* genes were located in the boundaries of LSC/IRb regions (JLB) consistently, except for the location of *rps19* from *F. esculentum* ssp. *ancestrale* in JLB was more forward than other members (1 bp). The SSC and IRb regions (JSB) were connected by *ndhF* genes, and the length of the *ndhF* in IRb from the JLB was 54–90 bp. In the JSA (SSC/IRa) regions, only JSA of three species were embedded in *rps15* gene, including the two *F. esculentum* and *F. luojishanense*. Specifically, the *rps15* gene was located on the right of the two *F. esculentum* with the distance of 2 bp, but that of *F. luojishanense* was 23 bp. The LSC/IRa (JLA) junctions in the cp genomes of 8 *Fagopyrum* species were identical. All in all, the IR boundaries of *F. tataricum* and *F. cymosum* were similar, as well as two *F. esculentum* species, and three wild species (*F. longistylum*, *F. leptopodum*, and *F. urophyllum*), respectively.

### Simple Sequence Repeats Analysis

**FIGURE 6:**
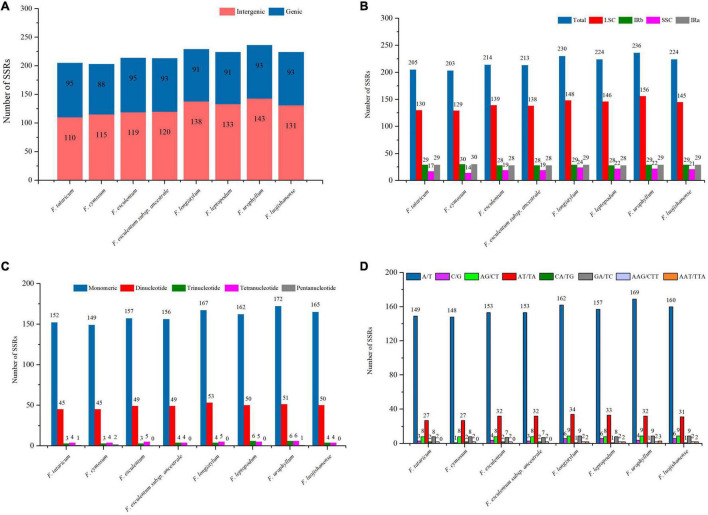
Analysis of SSRs in the eight *Fagopyrum* species cp genomes. **(A)** Distribution of SSRs in genic and intergenic. **(B)** Distribution of SSRs in LSC, IRa/IRb, and SSC. **(C)** The number of different SSR types detected in the eight *Fagopyrum* species cp genomes. **(D)** The number of different base SSR types in the eight *Fagopyrum* species cp genomes.

Simple sequence repeats, also known as microsatellites, consisted of short tandem repeats of 1–6 bp in length (Li B. et al., 2020). SSRs are widely distributed in the cp genome, and play a key role in the identification of plant genetic relationships and taxonomic status (Yang et al., 2019; Li Y. et al., 2020). In the cp genome sequence of the eight *Fagopyrum* species, SSRs were mainly located in the intergene region (∼57.72%), followed by the genic region (∼42.28%), while no SSR was observed in tRNAs and rRNAs (Figure 6A and Supplementary Table 3), which is consistent with the report of Wang et al. (2017b). Of note, the SSR numbers of *F. leptopodum* (133, ∼59.38%), *F. longistylum* (138, ∼60.26%), *F. luojishanense* (131, ∼58.48%), and *F. urophyllum* (143, ∼60.59%) in the intergene region were significantly higher than that of *F. tataricum* (110, ∼53.66%), *F. cymosum* (115, ∼56.65%), *F. esculentum* (119, ∼55.61%) and *F. esculentum* subsp. *ancestrale* (120, ∼56.34%). Most SSRs were located in LSC region (∼64.63%), followed by IR region (∼26.38%) and SSC region (∼8.99%) (Figure 6B and Supplementary Tables 4, 5). *F. cymosum* (129, ∼63.55%) had the least number of SSR in LSC region, followed by *F. tataricum* (130, ∼63.41%), *F. esculentum* (139, ∼64.95%) and *F. esculentum* subsp. *ancestrale* (138, ∼64.79%), in general, their number was significantly lower than *F. leptopodum* (146, ∼65.18%), *F. luojishanense* (145, ∼64.73%), *F. longistylum* (148, ∼64.35%), and *F. urophyllum* (156, ∼66.10%). Interestingly, as two typical cultivars, *F. tataricum* (58, ∼28.29%) and *F. esculentum* (56, ∼26.17%) showed significant expansion in SSR proportion in IR region. Further, a total of 24 gene located in different regions were found, which may be the result of co-evolution of cp genomes (Zhao et al., 2021). Among them, *ndhB*, *ycf2*, and *ycf1* are in the IRb/IRa region, *atpA*, *rbcL*, *rpl20*, *rpl22*, *rpoA*, *ycf4*, *cemA*, *petB*, *ycf3*, *petA*, *rpoB*, *atpF*, *rpoC1*, *rpl16*, and *rpoC2* are located in LSC region, and *rps15*, *ndhF*, *ndhD* are located in SSC region.

The distribution range of SSRs ranged from 8 to 16 bp in eight *Fagopyrum* species, with a total of 66 different types(Figure 6C and Supplementary Tables 4, 5). There were no hexanucleotide repeats have been found in these SSR sequences, and pentanucleotide repeats were only found in the cp genomes of *F. urophyllum* (ATTAT), *F. tataricum* (TTTTA), and *F. cymosum* (TCTAT/TTTTA). Among all *Fagopyrum* species, the number of mononucleotide repeats in the cymosum group was significantly lower than that in the urophyllum group. In general, this study supports that mononucleotide repeats may play a more important role in genetic variation in buckwheat than other SSR types (Huang et al., 2017; Liang et al., 2020). Although the chloroplast evolution of *Fagopyrum* species were relatively conserved, the cymosum group may be subjected to stronger selection and evolutionary pressure, resulting in the decline of SSR genetic diversity. Meanwhile, the number and types of SSR of the eight buckwheat plants in this study were further analyzed (Figure 6D and Supplementary Tables 5, 6). Further, the proportion of mononucleotide repeats for A/T and C/G types were 71.52 and 1.86%, respectively (Figure 6D and Supplementary Tables 5–7). This is similar to Zingiberales, Salicaceae, and Ranunculaceae, etc., indicating that mononucleotide repeats of A/T type may always be the most abundant base of simple repeat sequences (Huang et al., 2017; Liang et al., 2020; Park and Park, 2021). In addition, the number of mononucleotide repeats of A/T types or C/G types in the cymosum group was significantly lower than the urophyllum group, indicating that the number of SSR may still be similar in different subgroups of *Fagopyrum* species. The dinucleotides of eight *Fagopyrum* species were divided into four categories, which showed differences in some gene regions and repeated fragments among different groups. For example, repeat sequences of AG/CT and GA/TC types do not differ significantly between the cymosum group and urophyllum group. However, the proportion of CA/TG repeats in the cymosum group (∼0.96%) was much higher than that in the urophyllum group (∼0.44%). Similarly, AT/TA type accounted for the highest proportion of all dinucleotides (∼14.16%), which further confirmed the activity of A/T base in the cp genome. In this study, *F. tataricum* (27, ∼13.17%)/*F. cymosum* (27, ∼13.30%), *F. esculentum* (32, ∼14.95%)/*F. esculentum* subsp. *ancestrale* (32, ∼15.02%) had similar AT/TA types in number and proportion, which supported their genetic relationship to a certain extent. In addition, nucleotide repeats of AAT/TTA type did not exist in the four species of cymosum group (0), while *F. longistylum* (∼0.87%), *F. leptopodum* (∼0.89%), *F. luojishanense* (∼0.89%), and *F. urophyllum* (3, ∼1.27%) had a similar proportion. Therefore, there may exist two divergent evolutionary directions between the cymosum group and the urophyllum group. These results suggest that SSR can be used to identify genetic diversity, study evolution and develop molecular markers in buckwheat.

### Phylogenetic Analysis of Eight *Fagopyrum* Species Based on cp Genome

**FIGURE 7:**
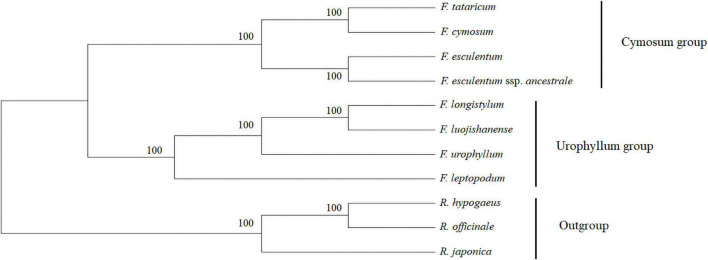
Phylogenetic tree obtained for eight *Fagopyrum* species inferred from ML analysis constructed by the complete chloroplast genomes. The number on the branches displayed the bootstrap support values.

Chloroplast genome sequences of eight *Fagopyrum* species and three Polygonaceae plants, which were selected as the outgroup, were used to construct phylogenetic trees to elucidate their genetic relationships (Figure 7). The numbers on the branches show the bootstrap value of the maximum likelihood analysis. The results showed that all *Fagopyrum* species clustered together at a very high resolution, and the three Polygonaceae plants and the eight *Fagopyrum* species were divided into two main types, which confirmed the independent differentiation status of the *Fagopyrum* from other genera of Polygonaceae. Further, eight *Fagopyrum* species were classified into two typical subclades. Among them, *F. tataricum* and *F. cymosum* formed a subgroup different from *F. esculentum*, which further supports that they may have a relatively high degree of homology and a closer genetic relationship. And then, they gradually converged with *F. esculentum* and *F. esculentum* subsp. *ancestrale* to form a subbranch. In addition, *F. longistylum* first approximates to *F. luojishanense*, and then gradually forms with *F. urophyllum* and *F. leptopodum*. These results showed that there might be two different subgroups among the eight *Fagopyrum* species, and the cymosum group and the urophyllum group evolved independently. Further, we developed six molecular marker sequences based on Pi values (Supplementary Figures 1A–F and Supplementary Table 8). And, six cluster trees were constructed based on these sequences using the neighbor-joining method (NJ). Among them, *trnS*-*trnG* and *trnV* trees supported the topological structure of the cp genome, which can be further applied in the identification of genetic relationships in *Fagopyrum* species.

### Phylogenetic Relationship Based on the ITS and *matK*

The most widely used chloroplast gene *matK* and nuclear marker ITS were selected to further speculate the genetic relationship of eighteen *Fagopyrum* species (including one variety: *F. gracilipes* var. *odontopterum*) (Supplementary Figures 2A,B and Supplementary Table 9). In general, the two ML trees based on ITS and *matK* supported the above-mentioned cp genome tree results: *F. tataricum* and *F. cymosum* in the two phylogenetic trees are first clustered into one branch, then clustered with *F. esculentum*, and then gradually clustered into other wild species. Therefore, phylogenetic trees based on different markers in this study all supported the conclusion that *F. tataricum* and *F. cymosum* in the cymosum group has a more close relationship than *F. esculentum*, which consisted with the previous study (Zhang et al., 2021a). Similarly, *F. luojishanense* and *F. longistylum* of the urophyllum group may be closely related, and then cluster with *F. leptopodum* and *F. urophyllu*m. These results further supported the chloroplast phylogenetic tree results. Therefore, the relationship of *Fagopyrum* plants was further inferred, *F. luojishanense*, *F. longistylum*, *F. gracilipes*, *F. gracilipes* var. *odontopterum* and other wild species may have a close relationship. According to the clustering results, *F. gracilipes* var. *odontopterum* as the division of *F. gracilipes* is considered reasonable. The *F. lineare* and *F. leptopodum* may be closely related to each other. They are both short plants, thin stem nodes, and highly adaptable in these *Fagopyrum* plants. Moreover, the two evolutionary trees supported *F. caudatum* and *F. pugense* were closely related. In general, these sequences of molecular markers with stable phylogenetic relationships of *Fagopyrum* plants will be considered as “references” to further infer taxonomic status among other species. However, it should be pointed out that the phylogenetic trees based on *matK* and ITS sequences could not completely define the relationships of some *Fagopyrum* species. For example, the genetic relationship between *F. macrocarpum* and *F. qiangcai* is still unstable. Therefore, it is necessary to further analyze the taxonomic status of *Fagopyrum* plants through extensive molecular marker sequences or complete genome sequencing.

## Discussion

### Sequence Differentiation

In this study, we compared the complete cp genomes of eight *Fagopyrum* species, which showed a typical circular tetrad structure. It consisted of a LSC region (84,494.9 bp in average), a SSC region (13,288.5 bp in average), and two reverse repeats (IR) regions (30,801 bp in average). The structures, genome lengths and proportion of these cp genomes were highly conserved. Among the eight cp genomes, the gene spacer is the largest variable region, which is consistent with most angiosperms (Wicke et al., 2011). The total GC ranges from 37.78 to 37.99%, which are higher than that of *Euonymus*, and *Curcuma* (Liang et al., 2020; Li et al., 2021). The GC ratios of the cp genome of angiosperms are usually between 34 and 40%, which plays an important role in the transmission of gene information (Zhu et al., 2017). The cp genome differences of different species are obvious through changes in base composition. These GC contents of the *Fagopyrum* species are the highest in IRa/IRb region, and the uneven distribution of GC ratio and gene conversion between IR sequences, which may be the reason why the IR region is more conserved than the LSC and SSC region (Khakhlova and Bock, 2006; Fan et al., 2018).

The contraction or expansion of the IR boundary is one of the main driving forces of cp genome length and structure difference, and the change of IR/SC connection location is a typical evolutionary phenomenon in plants (He et al., 2017). Interestingly, we found significant expansion of the LSC region in *F. esculentum* and *F. esculentum* ssp. *ancestrale*, which may be direct evidence of both cp genome length expansion and IRb region contraction. In addition, a significant contraction was observed in the SSC region of *F. luojishanense* (∼13,094 bp), which had the largest IRa/IRb region (∼30,870 bp), resulting in the C terminal of *rps15* crossing into the IRb region (∼23 bp). Furthermore, we found that the loss of functional genes in cymosum members were significantly higher than that in urophyllum group. And, this phenomenon was more obvious in many transfer RNAs. Therefore, we hypothesized that this deletion may result from the apparent activity of the highly structured chloroplast genome in cymosum group. For example, *trnfM-CAU* lost in *F. esculentum* and *F. esculentum* ssp. *ancestrale*. The chloroplast genome structures of urophyllum members were more conserved, and there were little difference in the numbers and positions of encoded genes. In addition, *trnfM-CAU*/*trnM-CAU*, *trnG-UCC*/*trnG-GCC* in cymosum group were significant differences in gene location in cp genomes. tRNAs are one of the most important and versatile molecules responsible for the maintenance and maintenance of protein translation mechanisms (Mohanta et al., 2019). Differences in the number and distribution of tRNAs in the cp genome may result in significantly influences of post-translational modification processes on genes in the photosynthetic system, especially *rpoA*, *rpoB*, and *rpoC* genes (Little and Hallick, 1988; Zhang, 2020). In addition, deletion of *rpl23* gene in cp genomes of two cultivated species (*F. tataricum* and *F. esculentum*) were observed. This phenomenon illustrated a typical case of protein (gene) substitution in the evolution of chloroplast ribosomes in *Fagopyrum* plants, and nuclear genome could progressively exert stronger over the chloroplast translational system (Bubunenko et al., 1994). It is worth noting that *F. esculentum*, as a *Fagopyrum* plant which is mostly distributed in the middle and high latitude areas of the northern hemisphere with long sunshine, is observed the most loss of functional genes, such as *trnT-UGU*, *rpl23*, *trnI-CAU*, etc.

### Divergence Hotspot Regions

DNA barcoding is widely used in species identification, germplasm management, genetic diversity analysis, phylogeny, and evolution (Gregory, 2005; Liu et al., 2019). In previous studies, the phylogeny of structural *Fagopyrum* plants was mainly based on SSR markers (Ma et al., 2009; Yang et al., 2020), single-copy nuclear gene (Ohnishi and Matsuoka, 1996; Ohsako and Ohnishi, 1998). The taxonomic analysis and genetic identification of *Fagopyrum* species are hampered by the lack of genomic information. Cp genome sequences are relatively conserved, which is less affected by non-parallel evolutionary in functional genes of nuclear genes in phylogenetic tree construction. Therefore, the cp genome sequences are often used in angiosperms phylogenetic prediction in recent years (Zhang et al., 2017; Zhao et al., 2020). To determine divergence packaging, the mVISTA program was used to compare the cp genome sequences of eight *Fagopyrum* species. The results showed that the cp genomes of eight *Fagopyrum* species were rich in the variable sites, and some regions with high variable frequency could be directly used as potential molecular markers for species identification (Song et al., 2017; Xu et al., 2017). In general, the proportion of variable loci in the non-coding region was higher than that in the coding region. Meanwhile, sequence differentiation in the IR region was slower and more conserved than that in LSC and SSC region. These results are consistent with most cp genome studies in plants, and we speculate that this may be due to higher gene conversion between the two IR regions (Khakhlova and Bock, 2006; Jansen and Ruhlman, 2012; Huang et al., 2014). In addition, the nucleotide diversity (Pi) of eight *Fagopyrum* species were assessed by sliding window analysis. These results of Pi values were generally consistent with mVISTA analysis, and the nucleotide diversity in the non-coding region was higher than that in the coding region. Six variable regions (*ndhF*-*rpl32*, *trnS-trnG*, *trnC*, *trnE-trnT*, *psbD*, and *trnV*) were identified as highly variable sites at the species level of *Fagopyrum*. These variable regions were further used to identify the genetic relationship of eight *Fagopyrum* species. And, the results showed that *trnS-trnG*, and *trnV* trees showed highly consistent results with cp genomes, so that they were further recommended as potential molecular markers in genetic development analysis and assisted breeding in *Fagopyrum* plants.

### Identification of Repeated Sequences

Simple repeat sequences play important role in the combination and arrangement of cp genome structures, which are highly variable in different species of the same genus, Thus, SSRs have been widely used in population genetics and species biodiversity studies (Thiel et al., 2003; Zhou et al., 2019). In this study, it was found that the SSR polymorphism levels of the four major components of these cp genomes were inconsistent. SSRs were mainly found in the LSC region of the eight *Fagopyrum* species, which was closely related to the interval length. The distribution density of SSRs in the eight *Fagopyrum* species were uneven, and there may be more SSRs in some sections and gene locus. For example, *matK*, *rpoC2*, *clpP*, *ycf1*, *ycf2*, *ycf3*, and other gene regions showed higher SSR density, which was consistent with Zingiberales and other plants (Liang et al., 2020). Although the cp genome evolution of *Fagopyrum* plants is generally co-evolutionary, some functional gene regions may respond to important biological effects and thus be subjected to more significant evolutionary pressures (Williams et al., 2019). At present, only a few “star genes,” such as *matK*, *rbcL*, *ycf1*, and *ycf2*, have been found as common positive selection sites (Liang et al., 2020; Li et al., 2021), other studies on the response evolution and biological role of chloroplast functional genes are still scarce. Nevertheless, it is desirable to select some segments or polymorphism of repeating sequence fragments from the cp genome as new tools for studying systematic differentiation.

A total of 110 (∼*F. tataricum*) ∼143 SSR markers (∼*F. urophyllum*) were found in the cp genomes of eight *Fagopyrum* plants, including mononucleotides, dinucleotides, tetranucleotides, trinucleotides, pentucleotide. Notably, there were no hexonotides found in all *Fagopyrum* species, which is inconsistent with Euonymus, Zanthoxylum, Curcuma, Wurfbainia Villosa, Amomum, Kaempferia, etc. (Liang et al., 2020; Li et al., 2021; Zhao et al., 2021). A/T and AT/TA repeats are the main SSR types, which may be because A/T bases are more easily changed than G/C bases (Li et al., 2021). However, these AT-rich regions did not contribute significantly to the expansion of cp genome size (Figure 6D). Compared with the gene regions, most of the SSRs were distributed in the intergene region (IGS region), which was more obvious in the members of the urophyllum group. It should be noted that there were significant differences in SSR markers in some gene regions between the urophyllum group and the cymosum group. For example, CA (4) existed only in cymosum group members, while AAT (4), AG (5), GA (5), TCAA (3), and TTA (4) were all found in urophyllum group members. These markers can be further applied to the identification of the two subgroups. In addition, many unique SSR markers were found in some *Fagopyrum* species, which can be used in the identification of different species. For example, AAAT (3) only existed in tartary buckwheat, AATT (4), A (16), TCTAT (3) only exist in *F. cymosum*, AATG (4) only existed in *F. longistylum*. Interestingly, there are still some unique SSR markers in *F. esculentum* and *F. esculentum* ssp. *ancestrale*, which will be effectively used in the identification of cultivated and wild ancestor species. For example, TTGA (3) was found in *F. esculentum*, while GTA (5), and C (12) were unique to *F. esculentum* ssp. *ancestrale*.

Interestingly, we observed significant differences in repeat sequences among some photosystem genes between members of the cymosum group and urophyllum group (Supplementary Table 7). For example, *ycf1* and two ribosome large subunit genes (*rpl32*, *rps15*) at the IR boundary showed significant SSR expansion in the cymosum group. This may contribute to the light adaptation of cymosum group members, which is conducive to planting (Fan et al., 2018; Liang et al., 2020). Photosystem subunit genes (*psaJ*, *psbK*, *psbZ*) showed significant SSR expansion in *F. esculentum* and *F. esculentum* subsp. *ancestrale*. They are more adapted to the long-sunshine of the northern hemisphere (Ikeuchi et al., 1991; Sugimoto and Takahashi, 2003). In addition, the urophyllum group members have a narrower distribution range, mainly growing in mountainous areas of southwest China. However, they are more adaptable to complex geographical environments, such as mountain areas and sandy areas, which are too harsh for the cultivated species (Zhou et al., 2018). In general, the process of artificial domestication or natural selection pressure leads to a significant decline in genetic diversity in the genome (Louwaars, 2018; Zhu et al., 2019). However, this was not significantly reflected in cp genomes of *F. cymosum*, *F. esculentum*, and *F. tataricum*. Therefore, we speculate that these domestication intervals may exist mainly in the nuclear genome. In conclusion, SSR markers of eight *Fagopyrum* species were systematically reported for the first time, which can provide a reference for the subsequent study of molecular evolution and phylogeny of *Fagopyrum* genus and Polygonaceae family.

### Phylogenetic Relationships

For a long time, the taxonomic status of *Fagopyrum* genus has changed frequently, and no consensus has been reached among different species (Linnaeus, 1753; Miller, 1754; Meisner, 1826; Gross, 1913; Stewad, 1930; Zhang et al., 2021b). In this study, the phylogenetic trees based on cp genomes of eight *Fagopyrum* species and *Rumex*, *Rheum*, and *Reynoutria* supported the independent evolution of *Fagopyrum* plants. Therefore, it is reliable that *Fagopyrum* has a separate taxonomic status in the Polygonaceae.

Furthermore, the cymosum members (*F. tataricum*, *F. cymosum*, *F. esculentum*, *F. esculentum* subsp. *ancestrale*) had significant independent cluster branches into the urophyllum group. Therefore, we infer that the evolutionary processes of the two groups of *Fagopyrum* species may be independent rather than overlapping. Similarly, the separation of the cymosum group and the urophyllum group may be earlier than the flower type differentiation of *Fagopyrum* plants, and then two pollination modes of self-pollination (self-compatibility) and cross-pollination (self-incompatibility) are produced. In addition, this study concluded that the genetic relationship in the cymosum group is clear, the *F. cymosum* and *F. tataricum* are more closely related than *F. esculentum*, although their pollination patterns are not consistent. However, the taxonomic status of the members of the urophyllum group are more complicated, as the urophyllum group consists of 18 species. Although there were significant differences in differentiation rates between nuclear and cp genomes, ITS clearly supported the clustering results of the urophyllum group in the evolutionary tree of cp genomes. Four urophyllum group members can further anchor the taxonomic status of other wild species members, which is further supported by the previous study (Cheng et al., 2020; Zhang et al., 2021a). It should be noted that the taxonomic status of some members of the urophyllum Group cannot be significantly anchored by a single molecular marker, which may require further molecular evidence.

## Data Availability Statement

The data presented in the study are deposited in the National Center for Biotechnology Information (NCBI) repository, accession number were: *F. longistylum* (OK054489), *F. urophyllum* (OK054490), *F. leptopodum* (OK054491).

## Author Contributions

KZ, MZ, JC, and YF conceived and designed the work. YT and MD collected the samples. YF, YJ, and KZ performed the experiments and analyzed the data. YF and YJ wrote the manuscript. MD, MZ, and JC revised the manuscript. All the authors have read and agreed to the published version of the manuscript.

## Conflict of Interest

The authors declare that the research was conducted in the absence of any commercial or financial relationships that could be construed as a potential conflict of interest.

## Publisher’s Note

All claims expressed in this article are solely those of the authors and do not necessarily represent those of their affiliated organizations, or those of the publisher, the editors and the reviewers. Any product that may be evaluated in this article, or claim that may be made by its manufacturer, is not guaranteed or endorsed by the publisher.

## Footnotes

1
https://chlorobox.mpimp-golm.mpg.de/OGDraw.html

## Supplementary Material

Supplementary Figure S1 Phylogenetic tree based on the *ndhF-rpl32* **(A)**, *psbD* **(B)**, *trnC* **(C)**, *trnE-trnT* **(D)**, *trnS-trnG* **(E)**, and *trnV* **(F)** sequences of eight *Fagopyrum* species constructed from NJ analysis.

Supplementary Figure S2 Phylogenetic tree based on the ITS **(A)** and *matK* **(B)** sequences of eighteen *Fagopyrum* species constructed from ML analysis.
