# Supplementary figures and images for: The Complete Chloroplast Genome Sequences of Eight Fagopyrum Species: Insights Into Genome Evolution and Phylogenetic Relationships
Source: Front Plant Sci. 2021 Dec 15;12:799904. doi: 10.3389/fpls.2021.799904 (PMC8715082; doi:10.3389/fpls.2021.799904)

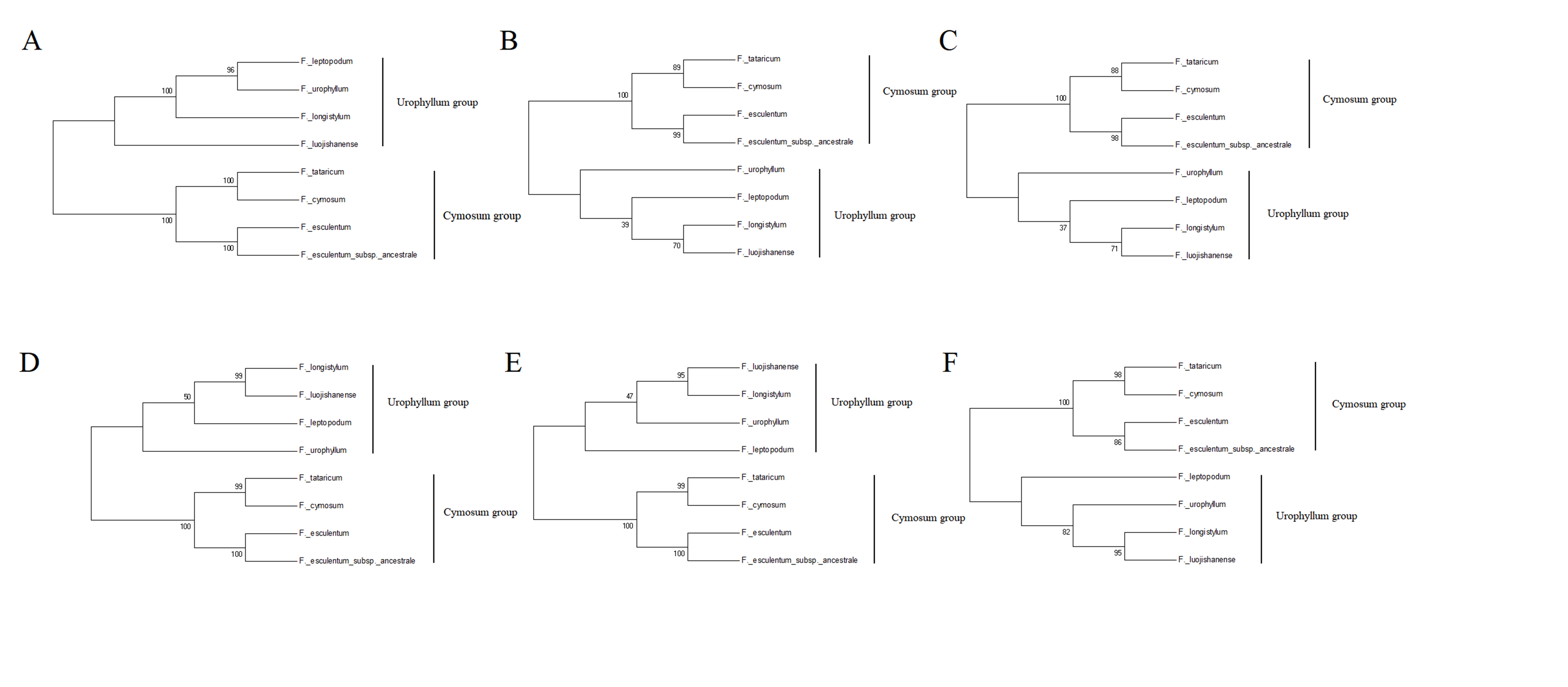

Supplement: Supplementary Figure S1 — Phylogenetic tree based on the ndhF-rpl32 (A), psbD (B), trnC (C), trnE-trnT (D), trnS-trnG (E), and trnV (F) sequences of eight Fagopyrum species constructed from NJ analysis. [file Image_1.TIF]

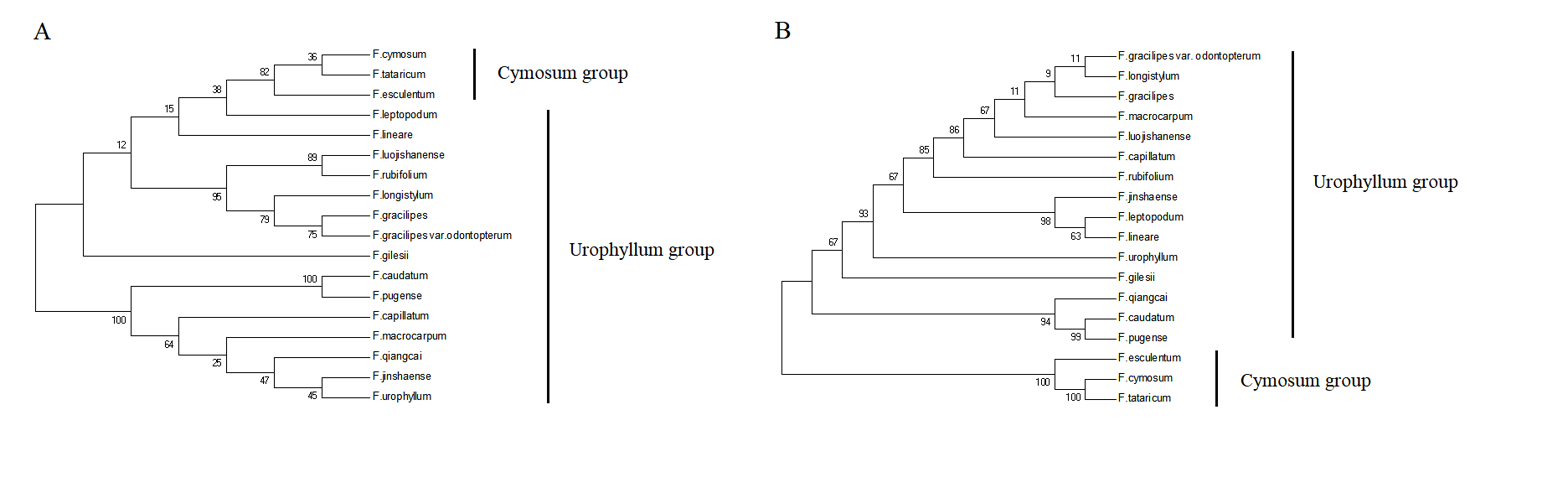

Supplement: Supplementary Figure S2 — Phylogenetic tree based on the ITS (A) and matK (B) sequences of eighteen Fagopyrum species constructed from ML analysis. [file Image_2.TIF]
